# Significance of intraoperative testing in right-sided implantable cardioverter-defibrillators

**DOI:** 10.1186/1749-8090-8-77

**Published:** 2013-04-11

**Authors:** Andreas Keyser, Michael K Hilker, Ekrem Ücer, Sigrid Wittmann, Christof Schmid, Claudius Diez

**Affiliations:** 1 Department of Cardiothoracic Surgery, University Medical Center, Regensburg, Germany; 2 Department of Cardiology, University Medical Center, Regensburg, Germany; 3 Department of Anesthesiology, Regensburg, Germany

**Keywords:** Implantable cardioverter defibrillator, Right-sided implantation, Intraoperative test

## Abstract

**Background:**

Implantation of implantable cardioverter-defibrillators (ICD) from the left pectoral region is the standard therapeutical method. Increasing numbers of system revisions due to lead dysfunction and infections will consecutively increase the numbers of right-sided implantations. The reliability of devices implanted on the right pectoral side remains controversially discussed, and the question of testing these devices remains unanswered.

**Methods:**

In a prospectively designed study all 870 patients (60.0±14 years, 689 male) who were treated with a first ICD from July 2005 until May 2012 and tested intraoperatively according to the testing protocol were analyzed. The indication for implantation was primary prophylactic in 71.5%. Underlying diseases included ischemic cardiomyopathy (50%), dilative cardiomyopathy (37%), and others (13%). Mean ejection faction was 27±12%. Implantation site was right in 4.5% and left in 95.5%.

**Results:**

Five patients supplied with right-sided ICD (13%, p = 0.02 as compared to left-sided) failed initial intraoperative testing with 21 J. 3 patients were male. The age of the patients failing intraoperative testing with right-sided devices appeared higher than of patients with left-sided devices (p = 0.07). The ejection fraction was 28±8%. All patients reached a sufficient DFT ≤ 21 J after corrective procedures.

**Conclusion:**

Implantation of ICDs on the right side results in significantly higher failure rate of successful termination of intraoperatively induced ventricular fibrillation. The data of our study suggest the necessity of intraoperative ICD testing in right-sided implanted ICDs.

## Background

Implantation of implantable cardioverter-defibrillators (ICD) in patients with high risk for life threatening ventricular arrhythmias is the standard therapeutical method [1]. The implantation of ICDs from the left pectoral region is accepted standard procedure, which has considerably improved over time by the development of new ICD-leads, shock algorithms, high energy defibrillators, and quick energy supply following the introduction of a new generation of capacitors ≥31 Joule (introduced 2005). Yet, pathological reasons (e.g. thrombosis, infection, abandoned leads) on the left side may force to implant the devices on the right side. With increasing implantations of ICDs the number of system revisions due to lead dysfunction and/or infections will rise and the number of right-sided implantations will increase consecutively. The reliability of devices implanted on the right pectoral side remains controversially discussed. The few studies reporting on right-sided implantations have unanimously found significant higher thresholds as compared to devices implanted left pectorally, but none of the studies have reported of failing initial intraoperative tests [2-8]. The present study evaluates intraoperative initial testing failure in right-sided implantations of ICDs in consecutive contemporary patients with a uniform protocol and arouses the issue of ICD testing in these patients.

## Methods

Since 1996, our institution implanted 1668 ICDs. With introduction of newly developed high energy devices (≥31 Joule devices) with quick energy supply the study was inaugurated 2005. To avoid bias of the study all patients requiring ICD therapy were included. Informed consent was obtained from each patient and approval of the institutional review board is given. Within the study period from July 2005 until May 2012, 876 patients received their first ICD (1-, 2-, and 3-chamber) devices.

### Exclusion criteria

Left ventricular thrombi were regarded as exclusion criterion to reduce the risk of embolization with respect to the intraoperative testing procedure. Patients requiring epicardial defibrillator patches also were excluded as they were not treated with endocardial defibrillator leads.

The primary endpoint of the study was patients failing the initial intraoperative testing of the system implanted.

### Surgery

All patients underwent general anesthesia for the operative procedure which was performed by a cardiac surgeon. Leads were placed via a cephalic vein and/or subclavian vein. Only dual coil defibrillation leads were implanted. Left ventricular leads were positioned either endocardially or epicardially. All devices were implanted subpectorally.

The devices were implanted on the left side in 831 (95.5%) of the patients. Due to occluded left subclavian vein or pacemaker on the right side 39 patients were treated with right sided devices.

All implanted systems were high energy ICDs with maximum deliverable shock energy ≥31 Joule (J) (Biotronic, Boston Scientific, Medtronic, and St. Jude Medical). Ventricular leads were placed in a right apical position, the distal end of the proximal coil near the atrium/superior caval vein junction. An adequate ventricular sensing of >6 mV and a pacing threshold of <1 V was attempted in all cases. Any antiarhythmic therapy was recorded.

### Testing

Initial testing was performed using active can and dual coil transvenuos lead. The superior caval vein coil and right ventricular coil was the cathode for the biphasic shock. According to the testing protocol ventricular fibrillation (VF) was induced by T-wave shock or - if T-wave shock failed to induce VF - by 50Hz pacing. The test shock was programmed at delivered energy of 21 J. The delivered energy of 21 J corresponds to a 10 J safety margin of a 31 J device. To avoid bias in the study, the 21 J margin was chosen, even if the delivered energy of an implanted device might have exceeded 31 J. In case of ongoing VF external defibrillation followed to terminate VF. If successful termination of VF could not be achieved with internal electrodes, the defibrillation lead was repositioned and testing was repeated, again with 21 J. Programming reversed polarity, single coil, and relocation of the device caudo-medially or laterally followed as further options if the system still failed to terminate VF, each step being tested again with 21 J.

### Statistical analysis

Statistical analysis was performed with Stata 10.1 SE for Windows (StataCorp, College Station, TX, USA). Continuous data were first tested for normality with the Shapiro-Wilk test and graphically with Quantile-Quantile plots. If normally distributed, these data are presented as mean (± standard deviation) or, if non-normally distributed, as median with interquartile range. Dichotomous data were expressed as numbers and percentages. Univariate comparisons were tested with Fisher’s exact test or the Chi-square test for categorical variables. The *t* test was used for continuous normally-distributed variables or Mann-Whitney’s test for non-normally distributed data. The tests were performed two sided and a p-value of <0.05 was considered statistically significant.

## Results

Due to left cavity thrombi four patients had to be excluded from the study. Another two patients requiring epicardial defibrillator patches also had to be excluded. In the investigated group of 870 patients operated within the last six years no major adverse events including death, stroke, and cardio-pulmonary depression occurred during implantation and testing. 39 patients had right pectoral implantation of their device (4.5%).

Overall, 38 (4.3%) patients had a DFT >21 J with the initial shock configuration and underwent system correction. The mean age of patients failing the initial test with left sided devices proved to be significant lower when compared to the age of the general study population (60±14 years versus 51±14 years - ranging from 22 to 71 years, p<0.0001). Mean age of the patients with right-sided devices and failing the initial intraoperative test was comparable to the study population, and higher when compared to left-sided devices failing the initial intaoperative test (left 49.6±14 years, right 61.5±7 years, p=0.07). There were 25 male and 13 female patients. The ejection fraction was comparable in both groups. The initial test was failed by a total of 38 ICDs, 33 were implanted on the left (87%) and 5 on the right side (13%). The failing rate of implanted devices on the right side appeared to be significant higher than devices being implanted on the left side (p=0.023). Demographic data are summarized in Table 1.

**Table 1:** Successful versus unsuccessful initial intraoperative testing

	**Left sided implantation**	**Right sided implantation**	**Total**
Successful [n; %]	798; 96%	34; 87%	832
Unsuccessful [n; %]	33; 4%	5; 13%	38
Total	831	39	870
		p-value (FET)	0.023

*FET* - Fisher’s exact test.

Of the 39 devices implanted right pectorally the indication for ICD implantation was prophylactic in 28 cases according to the MADIT criteria [9]. The underlying diagnosis of non-ischemic dilated cardiomyopathy was found in 16 of the 39 patients, the remaining 23 patients suffered from ischemic cardiomyopathy. Underlying diseases of the patients and indication for device implantation are summarized in Table 2.

**Table 2:** Baseline characteristics

**Variable**	**Left sided implantation**			**Right sided implantation**		
	**Successful [n=798]**	**Unsuccessful [n=33]**	**p-value**	**Successful [n=34]**	**Unsuccessful [n=5]**	**p-value**
Age [years]	60 ± 14	50 ± 14	< 0.0001	63 ± 13	62 ± 7	0.77
Gender						
Males	637	22	0.08	27	3	0.57
Females	161	11		7	2	
Ejection fraction [%]	27 ± 12	24 ± 12	0.15	26 ± 12	28 ± 8	0.72

Comparisons. Age: Unsuccessful left versus Unsuccessful right: p=0.07.

We supplied 16 patients with a VVI system; three of them presented with chronic atrial fibrillation. A DDD-system was provided in 16 patients: 6 of them suffered from sick sinus syndrome, 6 from intermittent atrial fibrillation, two patients from AV block II type Mobitz and AV block III, respectively. Seven patients were treated with cardiac resynchronization therapy (CRT-D) due to additional left bundle branch block (Table 3). Right ventricular sensing was adequate in all patients with 10.8±3 mV. The pacing threshold was 0.6±0.2 V/0.5 ms. None of the patients received amiodarone or any other antiarhythmic therapy at the time of implantation.

**Table 3:** Indications for the implantable defibrillator (ICD) implantation

	**Left implantation**			**Right implantation**		
	**DCM**^**A**^	**ICM**^**B**^	**Other**^**C**^	**DCM**	**ICM**	**Other**
Successful [n; %]	289; 93%	404; 98%	105; 100%	13; 81%	21;91%	0
Failure [n; %]	23; 7%	10;2%	0; 0%	3; 19%	2;9%	0
	p < 0.0001 (FET)			p = 0.63 (FET)		
	Primary^D^	Secondary^E^		Primary	Secondary	
Successful [n; %]	566; 96%	232; 97%		26; 77%	2;40%	
Failure [n; %]	25; 4%	8; 3%		8; 23%	3; 60%	
	p = 0.69			p = 0.12		
	VVI^F^	DDD^G^	CRT^H^	VVI	DDD	CRT
Successful [n; %]	496; 97%	204; 94%	98; 96%	14; 88%	13; 81%	7; 100%
Failure [n; %]	17; 3%	12; 6%	4; 4%	2; 12%	3; 19%	0, 0%
	p = 0.34 (FET)			p = 0.82 (FET)		

FET – Fisher’s exact test.

^A^DCM – Nonischemic dilatative cardiomyopathie.

^B^ICM – Ischemic cardiomyopathy.

^C^Other – Hypertrophic obstructive cardiomyopathy, amyloidosis, fibrosis.

^D^Primary – Primary prevention indication.

^E^Secondary – Seconardy prevention indication.

^F^VVI One chamber ICD.

^G^DDD Dual chamber ICD.

^H^CRT Cardiac resynchronization therapy.

All patients successfully reached a DFT ≤ 21 J (that is a safety margin > 10 J) by changing the position of the ICD-lead, replacing the device and/or optimizing the shock configuration (Table 4).

**Table 4:** Corrective measures in patients with primary right sided implantation

**No**	**Age [years]**	**Gender**	**Pace/sense**	**Lead reposition**	**Dual → single coil lead**	**Reverse polarity**	**Device reposition**	**Total no of shocks**^**A**^
1	50	Male	0.5 V; 0.5 ms/14 mV	1	1			5 (2)
2	70	Male	0.7 V; 0.5 ms/11 mV	1				3 (1)
3	64	Female	0.4 V; 0.5 ms/16 mV	1		1	1	7 (3)
4	63	Male	0.9 V; 0.5 ms/10 mV	1				3 (1)
5	61	Female	0.7 V; 0.5 ms/15 mV	1			1	3 (1)

^A^No of intraoperative shocks; (no of external defibrillations).

## Discussion

As the implantation of ICDs from the left pectoral region is accepted standard procedure, right sided implantations remain an exception [2-8]. A glimpse into the future unveils an increasing number of system revisions due to lead dysfunction and infections [10]. Consecutively the number of ICDs implanted in the right pectoral region will increase, and the question of intraoperative testing in right-sided devices will gain a new and more important quality.

Significant higher thresholds of right pectorally implanted ICDs as compared to left-sided implantations have been reported, and most of the literature was published prior to the introduction of high energy devices with a new generation of capacitors with quick energy supply [2-8]. A failing of intraoperative testing has so far explicitly only been discussed in a case report [11].

Only the determination of the DFT or a safety margin of 10 J tested intraoperatively indicates appropriate function of the implanted system. Furthermore, the detection of ventricular arrhythmias (VAs, e.g. ventricular tachycardia and/or fibrillation), lead function, and system integrity are checked while testing, latter to detect the rare case of device failure [12]. The aim of a safety margin of 10 J – as in our study – lowers the probability of retesting. In addition, testing a 10 J safety margin tends to have enough evidence and is generally accepted [13].

In our study, designed to include all consecutive patients (all comers), we found a failing first shock rate of 13% in patients having their device implanted right pectorally. An inadequate safety margin of less than 10 J is found in 6.2% up to 17% of patients receiving an ICD (left sided) [14,15]. In left pectorally implanted ICDs, lead repositioning, device relocation, additional leads, and changing device polarity are recommended to avoid insufficient therapy, e.g. failing of necessary cardioversion and/or defibrillation, if the intraoperative test fails [16-18].

In the study of Kirk [7] one fourth (25) of the patients received right-sided implants of ICDs. The study population is rather small. Only one patient failed adequate intraoperative DFT-testing. Neither Gold [8] nor we experienced such a high amount of patients presenting contraindications for left-sided implants. As the populations obviously differ concerning indication to right-sided implantation they seem not to be comparable.

Gold [8] used a DFT-protocol not being completed in three patients (7.3%) with right-sided implants because of high thresholds. Yet Gold does not describe alterations of the implants to achieve appropriate intraoperative testing results. And he found a near doubling of the mortality rate among patients with right-sided implants in his follow up. The question arouses whether there might be a link between testing failure and mortality in the study of Gold. We tested a safety margin, having found an initial failing of the intraoperative test in 5 patients. All these five patients achieved an appropriate safety margin of ≤21 J by altering the ICD system implanted (see Table 4).

An adequate ventricular sensing of >6 mV (10.8±0.3 mV) and a pacing threshold of <1 V (0.6±0.2 V/0.5 ms) was achieved in all patients, prior to testing. As all our patients achieved these values we must question whether right ventricular stimulation threshold alone has enough evidence for appropriate device function [19]. Five of our 39 patients had corrections of their implanted devices due to failing shock at the first attempt. Thus, our data will not strain the conclusion to refrain from intraoperative testing as a consequence of an adequate right ventricular threshold when implanting devices right pectorally.

Four of our 39 patients failing the initial intraoperative test had pacemaker leads implanted prior to the ICD implantation. The abandoned right ventricular lead of three of our six patients with right-sided devices might have had an impact on the testing procedure, even though Glikson has shown no increased risk of abandoned right ventricular leads in patients being supplied with left-sided ICDs [20].

Amiodarone is reported to have an impact on failing therapies in patients supplied with an ICD [14,21]. Napp therefore recommends intraoperative ICD testing in these cases [21]. As Napp especially recommends testing in young patients treated with amiodarone, and as Napp questioned the need of intraoperative testing in his title we must stress, that in absence of medication with amiodarone abstaining from testing cannot be recommended for right pectorally implanted ICDs. None of our patients failing the initial intraoperative test with right sided devices was treated with amiodarone or any other antiarhythmic medication perioperatively.

The need of cardioversion and/or defibrillation might be necessary as well when antitachycardia pacing (ATP) as alternative therapy of VAs for these severe arrhythmias fails [22]. Even if up to 80% of VAs may be treated sufficiently with ATP, this different mode of therapy cannot justify arguing against intraoperative testing, as Viskin concludes [23].

The main arguments against intraoperative testing are major adverse events such as intractable VF, hemodynamic deterioration, neurological impairment, and death [24-26].

Death in accordance to defibrillator testing has a prevalence of 0.016% in the Canadian population of 19067 patients through 0.2% of 440 patients published by Alter, and 0.4% stated by Kolb [24,25,27]. Strokes have been observed in 0.026% in the Canadian study through 0.5% by Alter [24,25]. The Canadian study is being multiply cited in the use for arguments against intraoperative testing. But Healey could verify these results in his study of 2173 patients finding no differences in the perioperative complications in patients having been tested or not [28]. In our total collective of 1668 patients treated since 1996 we observed no stroke and no deaths related to intraoperative testing. An adequate surgical implantation time as well as a strict agreement in the operating theatre concerning therapeutically procedures in case of intractable VF enhances the safety of a patient.

Stickberger *et al*. assumed an efficacy of about 5% when additional modifications of the implanted systems are necessary to achieve an adequate DFT [29]. Our system revisions after failed initial testing, however, were uniformly successful.

Finally, Strickberger states that the elimination of DFT or safety margin testing offers the opportunity to treat more patients with ICDs [29]. But would we willingly accept a high failing rate in our patients, especially when implanting devices right-sided? Even if ICDs represent a life-saving tool in the therapy of sudden onsets of VAs physicians should not abandon the standard of care for their patients.

## Conclusion

The development of new ICD-leads, shock algorithms, high energy defibrillators, and quick energy supply has improved the ICD devices. Nevertheless, the implantation of ICDs on the right side results in significantly higher failure rate of successful terminating intraoperatively induced ventricular fibrillation. Alterations of the implanted system such as lead repositioning, device relocation, and changing device polarity are effective means to achieve sufficient sensing of VAs and an appropriate safety margin to terminate life threatening VAs. The data of our study strongly suggest the necessity of intraoperative ICD testing in right-sided implanted ICDs.

## Abbreviations

ATP: Antitachycardia pacing; AV block: Atrioventricular block; CRT-D: Cardiac resynchronization therapy -defibrillator; DCM: Dilative cardiomyopathy; ICD: Implantable cardioverter defibrillator; DFT: Defibrillation threshold; DDD: Duel chamber; EF: Ejection fraction; ICM: Ischemic cardiomyopathy; J: Joule; ms: Millisecondes; mV: millivolt; V: Volt; VA: Ventricular arhythmia; VF: Ventricular fibrillation; VVI: One chamber.

## Competing interests

The authors declare that they have no competing interests.

## Authors’ contributions

AK is the guarantor of the manuscript. AK contributed to the study conception and desig; acquisition, analysis and interpretation of data; drafting the article; and revising, reading, and approving the final version of the article. MKH contributed to conception and design and helped draft the manuscript. EÜ contributed substantially to the design and to the interpretation of data. SW helped draft the manuscript and contributed to the interpretation of data. CS contributed to conception and design of the study, contributed to the interpretation and helped draft the manuscript. CD helped design the study, contributed to the conception, performed the statistical analysis and helped draft the manuscript. All authors read and approved the final manuscript.
